# Surgical Sketches

**Published:** 1852-12

**Authors:** W. E. Horner

**Affiliations:** Prof. of Anatomy in the University of Pennsylvania, Senior Surgeon at the St. Joseph’s Hospital, &c. &c.


MEDICAL EXAMINER,

AND

RECORD OF MEDICAL SCIENCE.

NEW SERIES.—NO. XCVI.—DECEMBER, 1852.

---

### ORIGINAL COMMUNICATIONS.

---

*Surgical Sketches.* By W. E. Horner, M. D., Prof. of Anatomy in the University of Pennsylvania, Senior Surgeon at the St. Joseph's Hospital, &c. &c.

*A Military Hospital at Buffalo, New York, in the year* 1814.

The concentration of numerous forces at this post early in the spring—the arrival of many officers of high grade—the accumulation of large amounts of military stores—the daily drills and parades lasting from eight to ten hours—the presence of Major General Brown in command, with the recently appointed Brigadiers General Scott and Ripley of the Regular Army, and the chivalrous General Peter B. Porter, of the New York militia, all led to the conviction that a great enterprize was at hand; and that the young medical aspirants of this division of the United States forces, and all of us who felt desirous of some degree of experience in surgical matters for our better instruction, were likely to obtain it before long. The cessation of the wars in Europe, by the abdication and downfall in April of the most renowned of moderns, the Emperor Napoleon, in leaving the

British veterans unemployed at home, was an assurance also that they would be brought into the field against us; and that a conflict of the most sanguinary kind for the numbers engaged was about to commence.

Each party sustained by its warrant of national bravery and enterprize, and stimulated by a belief of wrongs unjustly suffered, felt the desire of redressing them through the ultima ratio on the field of battle. On the one side, we had the institutions of our country to make good against an enemy of all others the most capable of prostrating them, and of reducing us once more to a colonial state. The Government of Great Britain, on the other side, had to assuage the recollections of the American Revolution, and to punish us for the audacity of a declaration of war against her, made at a time when she was too much employed in Europe to direct any large division of her forces upon us.

It was evident that a struggle of no ordinary might was at hand. The skill, the confidence, and the steadiness of the heroes of the Peninsular campaigns, who had fought under Wellington, were to be tried against the ardor, the impetuosity, and the devotion to country of American soldiers. Laurels gained were to be in the same field against laurels in hope or desire. The tramp of battalions, the display in line, the practised fusillade, the dashing evolutions of artillery, the mimic charges of cavalry, the shrill penetrating word of command as it rang through the air,* all served to keep up the military animation now glowing in the bosoms of the American columns.

*General Scott's voice at this time was as remarkahle for its military tone, as his bearing and person were for their military contour. His stature is so well known in the United States, that it is scarcely necessary to say that he stood upwards of six feet. This favorite of that day with both soldiers, officers and the community, had risen very rapidly from the commission of Capt. of Artillery to the rank of Brigadier General. Opening his career of distinction at the battle of Queenstown Heights in 1812, as Lieut. Col., his valor on the occasion, and in the campaign of 1813, when Toronto (then Little York) and Fort George were taken, marked him out as one of the master spirits of the time. In 1814 he therefore appeared at the head of a brigade, and as the right arm of Major General Brown, an officer of great energy, but less practice than General Scott.

Such has been the rapid progress of our country in all the elements of power and of greatness since that period, that now are scarcely to be realized the difficulties and discouragements of its position then. It was at that period a much more responsible affair for the infant power of the United States to take a hostile attitude against the matured and colossal one of Great Britain than at present. This, however, is said in the most friendly spirit, and with a full desire that empires so capable of mutual injury and of disturbing the repose of the entire world, may both have the good sense and the high moral tone, to cultivate to the utmost reciprocal good will, and the commercial interchange of objects of national skill and industry, upon which the prosperity and happiness of both countries so essentially depend.

In the year 1813 I had seen but little service, my commission as Hospital Surgeon's Mate, dated July 3d, and signed by Gen. Armstrong, Secretary at War, having come to hand too late for any of the important field operations. The town of Little York, Upper Canada, had been taken, April 27th, and Fort George, at the foot of the Niagara River, on May 27th. The former with a very heavy loss, principally from the explosion of a British magazine; among the victims to which was General Zebulon Pike, the most promising officer of the time.

The American force at York consisted of about seventeen hundred men, and the British of about eighteen hundred. The former lost three hundred and twenty in killed and wounded, the latter about four hundred. Dr. James Mann,* Hospital Surgeon being attached to the expedition, says that the American column halted at a distance of four hundred yards from the enemy's batteries to reconnoitre, and that at this moment the explosion occurred, whereby sixty rank and file were killed, and one hundred and eighty wounded and mangled in a most wretched and deplorable mannner, by the fall of stones which formed the magazine.

The attack on Fort George commenced by a heavy cannonade on the 25th and 26th of May, from Fort Niagara and batteries recently erected, and the assault was made on the 27th. In the attack on Fort George, the Americans had about four thousand troops, of which twenty-seven were killed and eighty-seven

*Medical Sketches, &c. p. 61. Dedham, 1816.

wounded. The British loss was estimated at one hundred and two killed and 175 wounded.*

The battle was fought near the shore of Lake Ontario. Dr. Mann, who was on the spot immediately after the action, says that he found on the high bank in a space of fifteen by two hundred yards near four hundred men either killed or wounded, the number being made up from both armies as they lay intermixed. The contest was evidently a very severe one.

Expeditions having a disastrous result were sent out afterwards, one to Stoney Creek, and another to Beaver Dam. In the first, the two general officers were taken prisoners, and the troops returned precipitately to Fort George. In the second, a brilliant command of six hundred men laid down their arms to a body of the enemy scarcely large enough to guard them, having been made by a course of strategy to believe that they were surrounded and on the eve of an attack from an overwhelming force.

Many capital operations both upon the wounded Americans, and English who were taken prisoners, resulted from these several actions.

On my arriving at Fort George in August 1813, the army was tranquil, and there was no special duty for myself. At that time, it had been determined that the forces should leave this position and make a descent upon Montreal.

The General Hospital was then at Lewistown under Dr. Mann. Nearly about this period more than one third of the soldiers of the army were on the sick report. Half of the medical staff attached to regiments were disabled. Of seven surgeons' mates belonging to the Hospital one died, three had leave of absence, and the other three were for a short period sick. At one time, with a sick list of some six or seven hundred men, only three surgeons were present for duty. The diseases were typhus and intermittent fevers, diarrhœa, and dysentery.†

From the preceding battles and from some skirmishes on the outposts, there remained many who, from the nature of their wounds, were incapable of further military duty.

A detachment of some seventy three such soldiers, of which I

*Mann, loc. cit. p. 62.

†Mann loc. cit. p. 66. et. seq. See appendix No. 1.

still have the original list, dated Sept. 20, and also the General Order directing their destination, was committed to my professional charge, under the command of Lieut. Whiting, 23d Infantry, with direction to take these "brave and unfortunate men" to Greenbush by Oswego, and that they be treated on the way with the utmost kindness and attention. Many of them had been most distressingly mutilated by the fall of stones at the explosion of the York magazine, and some still required surgical dressing. Military orders are of obligation, and the execution of them was instituted forthwith.

Dr. Mann. loc. cit. p. 94, has inserted the name Hugo, and there is also some mistake about the officer in command, it, according to him, being Lieut. Archer, regimental pay-master, instead of Lieut. Whiting. He may be in one sense correct, that the officers alluded too were possibly detached originally for the duty; but the documents in my hands show the persons who executed it to have been as stated by myself. The following general order and list of the invalids referred to is inserted, as upon it there may be names interested in the recent distribution of public lands to the soldiers of that period, and where the muster rolls and other authentic documents of service may have been lost, owing to the destruction by the enemy of the public archives at Washington by fire, the subsequent season.

*Report of wounded soldiers, subjects for furloughs in the General Hospital, at Lewistown, Sept. 20th,* 1813.

| No. | Names. | Reg't. | |
|---|---|---|---|
| 1. | Jeremiah McDonald,—contracted hand, | 15 | |
| 2. | James McMinus,—fractured leg, | 6 | |
| 3. | Jacob Writer,—amputated leg and arm, | 15 | |
| 4. | William Hommy,—fractured leg, | 15 | |
| 5. | William Brady,—amputated arm, | 2 | Artillery |
| 6. | George Walton,—amputated leg, | 15 | |
| 7. | John Leech,—wounded in the jaw, | 6 | |
| 8. | George H. Clair,—wounded in the foot, | 15 | |
| 9. | Abell Gussum,—amputated arm, | 21 | |
| 10. | Loran Pottle,—amputated arm, | 3 | Artillery |
| 11. | Ransum Mix,—amputated leg, | 6 | Artillery |
| 12. | William Cooney,—amputated leg, | 22 | |
| 13. | William Pinter,—wounded in the thigh and knee, | Rifle corps. | |
| 14. | Alexander Frazier,—wounded through the shoulder, | Rifle corps. | |
| 15. | Harvey Johnson—wounded through the knee, | 21 | |

| No. | Names. | Reg't. | |
|---|---|---|---|
| 16. | James Hull,—fractured arm, | 21 | |
| 17. | Christian Shoemaker,—wounded in the back, | 16 | |
| 18. | Elijah Blodgett,—wounded through the neck, | 23 | |
| 19. | John Lawless,—wounded back and arm, | 14 | |
| 20. | Robert Davis,—wounded in the arm, | 14 | |
| 21. | Henry Corpe,—wounded in the wrist, | 14 | |
| 22. | Adam Feather,—wounded in the hand, | 22 | |
| 23. | John King,—wounded in the thigh, | 2 | Dragoons. |
| 24. | James Andrews,—wounded in the leg and thigh, | 2 | " |
| 25. | Lewis Myers,—broken arm, | 2 | " |
| 26. | Henry Renchert,—wounded in the hand, | 13 | |
| 27. | James H. Nevers,—wounded in the thigh, | | Rifle Reg't |
| 28. | Guy L. Carpenter,—wounded in the shoulder, | | " |
| 29. | Edmund McKenny,—wounded thro' both thighs, | | " |
| 30. | And'w McGenly, wounded in thigh, and bad enough | 14 | |
| 31. | Jacob Dullas,—loss of eye sight (purblind.) | 15 | |
| 32. | Peter Robbins,—wounded stiff knee, | 2 | Dragoons. |
| 33. | Jonas Horrington,—wounded in the shoulder, | 2 | " |
| 34. | Thomas Hennessey,—wounded in the arm, | 2 | Artillery. |
| 35. | Sergeant Nichols,—wounded in the shoulder, | 3 | " |
| 36. | Sergeant Smith,—wounded thigh and shoulder, | 2 | " |
| 37. | Patrick Mooney,—amputated arm, | 6 | " |
| 38. | Timothy Ford,—wounded in the wrist, | 2 | " |
| 39. | Sergeant Norton,—amputated leg, | 14 | " |
| 40. | Thomas Broughton,—caries of the jaw bone, | 6 | " |
| 41. | Samuel Benedict,—amputated arm, | 23 | " |
| 42. | James Evens,—wounded leg, | 15 | |
| 43. | Sergeant Brown,—wounded in the thigh, | 22 | |
| 44. | David Parson,—amputated thigh, | | Vol. corps. |
| 45. | James Martial,—wounded, | 23 | |
| 46. | Wellehemius Slighter,—wounded, | " | |
| 47. | Lewis Rickmon, " | " | |
| 48. | Jonathan Patt, " | " | |
| 49. | Leban Cooper, " | " | |
| 50. | Daniel Brown, " | 13 | |
| 51. | John Rengleher, " | " | |
| 52. | Abel Parker, " | " | |
| 53. | Lewis Jones, " | " | |
| 54. | Eastman Corbin, " | 25 | |
| 55. | Waterman Horris, " | 1 | Artillery. |
| 56. | Frederick Campbell, " | | " |
| 57. | Gian Lazino, " | | " |
| 58. | Thomas Moore, " | | " |
| 59. | John Lesso, " | | " |
| 60. | Nicholas Welch, " | | " |
| 61. | Jeremiah Nicholas, " | 3 | Artillery. |
| 62. | Benjamin Shearman, " | 14 | |
| 63. | John Clemmons, " | | |

| No. | Names. | | Reg't. | |
|---|---|---|---|---|
| 64. | James Conner, | wounded | | |
| 65. | Allin Rell, | " | | |
| 66. | James Nicholas, | " | | |
| 67. | Joseph Webb, | " | 15 | |
| 68. | Samuel Palmer,—amputated thigh, | | 2 | Artillery. |
| 69. | John Loudeman,—wounded, | | | " |
| 70. | Joseph Davis, | " | | " |
| 71. | John Beard, | " loss of fingers, | | " |
| 72. | Thomas Borden,—wounded in the hand, | | 15 | " |
| 73. | William Pringle,—discharged. | | | |

Greenbush was reached only on the 5th of November, and all further campaigning for the season was lost to the writer. This disappointment being unavoidable, it was hoped to retrieve in the present campaign (1814) the advantages thus missed.

Two brigades of regulars, one battalion of artillery, and, to which may be added, a brigade of militia volunteers and of Indians, under General Peter B. Porter, constituted the force of our army, amounting in all to four thousand men, or near it. This statement might be superfluous, but as the American force never exceeded that during the most active period, it exhibits a relation of force with that of wounded, which is of some importance. The generation which saw that array is now well passed away. It is only a few months ago that we read, as one of the remarkable occurrences of the time, that one hundred and fifty of the veterans of the war of 1812, had assembled in New York on some public occasion.

To the existing generation, who know Buffalo as it is, to wit, a well built, dazzling and opulent city, of from fifty to sixty thousand souls, with all the luxuries of modern times in it, its state at that period may be almost incredible. It has now an extensive mole, with a spacious harbor, which witnesses the hourly arrival and departure of magnificent and enormous steamers. At that time its creek was its only harbor, and that exhibited merely a few batteaux lazily drawn up along its shore, or some few canoes of the Indian tribes living in its vicinity. The place had literally no harbor which could be called such; the great Erie Canal was simply in cogitation, and the term rail road was unintelligible. The whole number of human habitations in the form of buildings was from eighty to ninety, small for the most part, and unfinished; there was among them but one brick house, known as Langdon's tavern, and the majority of the buildings were what were called shanties. They were very much in the style of hog pens, with board tops, and their destination was to retail whiskey to the soldiers.

It is true Buffalo had been entirely burned up the preceding winter, but its population before then scarcely exceeded a thousand people, and the houses were indifferent wooden structures, from one hundred to two hundred in number. A thick forest, with its primitive growth, almost unmolested, and impenetrable, except by a bad, miry road, led from Buffalo to Black Rock.

They were both villages, and in a state of rivalry, as far as such a thing could go. Black Rock had the advantage of being the natural harbor for the lower end of Lake Erie, but as its shores were within short cannon shot of the British batteries on the other side, it afforded no real protection, except from the lake storms, less injurious at that time than the showers of balls. It had about ten houses in it. Lewistown, as Dr. Mann says, was a handsome site for a town, "the name of which it only bears," for it consisted of a few *log* houses.

The Falls of Niagara were in a primeval state almost, the only marks of civilization being an old saw mill at Fort Schlosser.

While I am on this subject, the facility of getting from Philadelphia to Buffalo may amuse younger readers. By leaving Philadelphia at 8 o'clock A. M., of one day, New York could be reached the next day at 11 A. M., by diligent travel, and sleeping at New Brunswick. Now, five hours accomplish the same distance. By leaving New York at 5 o'clock P. M., in the best steamer, and going all night, Albany was reached at 7 P. M. the next day. It took eighteen hours of constant travelling to go from Albany to Utica, and three days of severe travelling to go from Utica to Buffalo, all of which was great expedition in its time. Thirty-six hours in all will now pass the traveller from Philadelphia to Buffalo, near six hundred miles; and if rail-way cars be comfortable to him, he may sleep away any amount of that time. Instead of having his joints, as formerly, half dislocated by log roads from Batavia to Buffalo, and his tranquillity interrupted by the frequent announcement,

"very miry here, gentlemen, be pleased to get out, unless you want to stick fast in the mud,"* he passes very rapidly from one place to the other.

These reminiscences, in marking the progress of our country, may surprise some of the younger members of the profession; they also have their use, though they may not be exactly surgical. They show the difficulties and expense of the transportation of troops and of munitions of war, to what was then an almost isolated and desolate frontier post.

In those days the communications of the country were so bad, that it is said every cannon conveyed from Albany to Sackett's Harbor, cost one thousand dollars, and that the "flour for General Harrison's army in the North-west, stood at one hundred dollars a barrel."†

The encampments of the army at Buffalo were broken up about the first day of July, 1814. Orders were issued for hospital preparations, a number of tents were left behind for future sick service, and for the sick of the regiments then on hand. The present Eagle Hotel and Rail Road Depot of Buffalo occupy the part of the city upon which the hospital was opened. The entire area allotted to it was to the west of the principal street, upon the first rise of ground there in ascending from the Creek. The space was about equal to that of the State House Square in Philadelphia, perhaps longer, in being more of an oblong.

While in the act of getting the hospital ready for service, it received a visit from General Scott, the universal favorite of the day, for his gallantry in the preceding campaign. As he rode through the hospital grounds, in his usual dashing style, with his aids, he said in passing, "Well, Doctor, but little work here as yet." "No, General, we are looking for some." "You will get it before long," was his reply, and off he careered with his staff.

* Dr. Mann, (Med. Sketches, p. 94,) says of this road: "From Batavia to the Niagara river, was a tedious journey of forty miles, for sick men, and these roads, bad at best," had been rendered almost impassable by heavy rains at the time he alludes to, October, 1813. Batavia then had forty houses.

† Ingersoll's Historical Sketch, &c., Vol. I., p. 283.

His promise was sufficiently kept, as the records of that celebrated period will show. It was the first time since the declaration of war that the tactics of an open field combat were tried. Line having been regularly displayed against line, each party directed by its special inspirations of skill and valor. There was, perhaps, never a campaign in which the belligerauts came to a better understanding of what they might expect in battle at each other's hands; and where the leaders, though under the excitement of a state of war, left off with more military respect for one another.

The operations commenced with the crossing of the Niagara river near its head, at Black Rock, by the American army, under the direction of Major General Brown. This was accomplished on the night of the second of July, and early the next morning, Fort Erie, nearly opposite the place of embarkation, was invested. A few scattering fires were directed from the Fort, and it surrendered in the afternoon of the third. In this affair only two or three soldiers were wounded, one in the knee, by a grape shot, and another in the head, by a buckshot.

The first one must have had his knee in a flexed position at the time of injury, judging from the course of the ball. The ball entered on the end of the right tibia, opposite its head; it did not penetrate or injure the bone, but glancing obliquely upwards, came out in the inside of the vastus internus, just above the knee.

From the nature of this wound, and the pain the patient experienced, an unfavorable result was looked for. The patient was dressed with a pledget of lint, and a bandage, on the field. The day afterwards, he was brought to the general hospital at Buffalo. I removed the first dressing, washed the wound well with soap and water, and applied a pledget of lint spread with simple cerate, and confined it with a bandage loosely applied. The use of ardent spirits, then universal, and really considered as the water of life, (aqua vitæ) was forbidden. He was ordered to live on thin soup and boiled rice, and to keep the limb undeviatingly in a straight position. On the fourth day after the injury, the pain of the limb increased, and a swelling of the joint was perceptible; the part was so extremely tender to the touch that the patient could scarcely bear the falling of the water on it from a sponge used in dressing it. A saturnine poultice was then applied; he was bled to the amount of a pint, and, in order to counteract the irritation of the wound, which had kept him sleepless since its reception, an opiate was given. On the morning of the fifth day the pain had abated in a measure, the swelling was stationary, and a small quantity of pus was perceptible on the surface of the wound. The poultice was renewed, and the opiate at night. This plan of treatment assuaged the violent pain; the sore got into a healthy condition on the tenth day. The suppuration became very copious and healthy, and the tension of the knee removed; everything was then dispensed with, except the daily washing of the sore and a dressing of cerate.

The suppuration gradually diminished, the cicatrix contracted, the knee became flexible, and, on the fortieth day after the reception of the injury, he returned to his duties in the line, in consequence of a general order for all convalescents of the hospitals, able to bear arms, to repair immediately to their respective corps.

The other patient, who was wounded in the head, was a boy of fifteen, much esteemed in his company for his gallantry and attention to his duties. He being on a scouting party, employed in exploring the adjacent country, the party was met at night in the woods by another of our scouting parties on the same business. They mistook each other for the enemy, and a firing ensued accordingly, in which one on each side was wounded, before they discovered their mistake. This boy was brought to the hospital the next day; he was in a comatose state, attended with delirium; however, when spoken to, his attention could be directed to the person who addressed him. The wound was extremely small, in consequence of being inflicted by a buckshot, was situated on the right temple, and had been closed up by the tumefaction of its edges, so that only a small bloody scab about a quarter of an inch in diameter, was visible. The temple was much swollen; he complained of great pain in the right ear and back of his head. The wound being closed, prevented the probing of it.

A poultice of bread and water (it being impossible to obtain milk,) was applied and confined by a bandage.

The patient, from his restless and painful situation, did not allow this to remain more than an hour or two; it was frequently applied, and as often displaced; it was given up the next day and the wound dressed with cerate. Third day, the appearance of the wound was not much altered, it had discharged a little blood and serum; the patient still restless, and moaning through excess of agony; his pulse was frequent and feeble. An anodyne at night. A little nourishment of soup was occasionally put into his mouth. The fourth and fifth day he was in pretty much the same situation as in the preceding, only it was more difficult to obtain his attention, the delirium and comatose state having increased. Death put an end to his sufferings on the morning of the fifth day.

On examining the head, it was found that the buck-shot had passed through the temporal muscle and entered the cranium through the anterior angle of the right os parietale, just before the squamous suture, penetrated through the dura mater into the substance of the brain, and passed through the cortical part of it, not far from the right lateral verticle, and lodged above the tentorium on the same side.

From the first day of July we had been busily employed, under the direction of Dr. William Thomas, Hospital Surgeon, in erecting hospital tents, procuring bunks and straw, and making every arrangement for the reception of a large number of wounded.

On the fifth of July the battle of Chippewa was fought, in which the first brigade, under General Scott, gained a complete victory over the enemy under General Riall.

Many prisoners, and most of the enemy's seriously wounded, fell into our hands, which, added to our own wounded, gave the surgeons of the hospital department as much business as they could well attend to. Many operations were performed on the field of battle, and all the wounded dressed there.

The entire loss in missing, killed and wounded, of the Americans, was estimated at three hundred and twenty-eight; that of the British was said to be much larger, owing to the effectiveness of the American musketry. The battle being fought on the banks of the Niagara river, the wounded were brought up in boats to the general hospital at Buffalo.

They were conveyed from the boats on Buffalo creek, to the hospital, a distanee of three or four hundred yards, on blankets, the sides of which were nailed to poles nine or ten feet long. This formed an easy and convenient litter, by which four strong men could safely convey one wounded, without exposing him to the unspeakable pain from jolts, &c., which would be the inevitable consequence of transportation by wheel carriages. Besides this advantage of the litter, when the wounded soldier was to be placed on it, it was spread smoothly on the ground and he slipped gently on. It was then taken up carefully by the assistants and carried to the hospital, when the patient was either assigned at once to his tent, or placed on the hospital parade ground, as the convenience of dressing required. A litter thus constructed can be easily pulled away from under the patient without pain, and is, in that respect, much better than the brancard or the handbarrow.

The wounded on this occasion, for the most part, had this second dressing on the hospital parade ground; as they were brought up from the boats, and the weather was remarkably fine. They were then sent to their tents respectively.

The number placed immediately under my charge amounted to from sixty to seventy. After the battle of the 5th, a still more desperate battle, that of Bridgewater, was fought on the 25th. The details of this fight are of a most singular kind. It was intended by the British Generals, Drummond and Riall, for the 26th, but by accident came off on the 25th. It was characterized at almost every period by the desperation marking the storming and defence of a fortified post. The American force, it is believed, mustered not more than twenty-five hundred fighting men. The British force being recruited during the battle, exceeded ours. The battle lasted for five hours, and ended at midnight. The British Major General Drummond was severely wounded—the second in command, General Riall, was made prisoner. Major General Brown was badly wounded—General Scott also. General Ripley's hat was shot through. Every officer almost in the American lines bore marks of his participation in it, either by wounds actually received in his person or in his clothes. As I saw the remnants of this gallant force, numerous were the cases of perforated hats—lacerated stocks—and gaping coats and vests; injuries which the difficulties of the times prevented their owners from readily repairing. Seventy-six officers, and six hundred and twenty-nine rank and file, were killed or wounded, of which number General Scott's brigade counted thirty-eight officers, and four hundred and sixty-eight rank and file. Of a force, then, of twenty-five hundred men, seven hundred and five were put *hors du combat* by the casualties of war, and it is thought that one hundred and fifty-five fell into the hands of the enemy.

The disorganization was so great from the loss of staff officers and soldiers, that it has been extremely difficult ever since to analyze this battle and show its precise results. The account published by the Hon. Charles J. Ingersoll,* corresponds more nearly with the camp rumors and conversations of the time, than any thing else I have seen, and it has perhaps the additional advantage of recurrence to original documents in the War Office, and of personal inquiry from the leaders on this celebrated occasion. It may here be suggested, should this medical sketch excite further interest as regards military matters, the same work may be very advantageously studied concerning the other operations on this frontier during the campaign of 1814. As to its precise accuracy, a verdict from other quarters will have more authority. One might suppose that in a military organization, every thing may be told with absolute accuracy, and this will be the case in a state of peace. But the tumult, confusion, and hurry of real military operations, and the want of even common personal or mechanical conveniences to make out reports and prepare records, produce to some extent an unavoidable inaccuracy. No man can see all at once even over a small space; he is therefore under the necessity of using the eyes and the testimony of others in the same difficulty with himself, and consequently aggregate results are the nearest approach to absolute facts.

Even in a military hospital, on such occasions, it is impossible to keep pace with the events in each ward or division of it. Much passes without being known at all—and much with being only loosely known. There are many occasions or periods in

* Historical Sketch of Second War, &c., chap. iv., Philadelphia, 1849.

which the mind is constantly engaged with present matters, and in thinking forwards; instead of thinking backwards, so as to collect, to compare, and to remember. A short lapse of time will obscure an impression or even efface it from the mind, and the advantage of it is thus sacrificed.

The British lost eight hundred and seventy-eight in killed, missing and wounded. All our serious cases of wounds were brought into the hospital at Buffalo; the lighter cases were left with the regimental hospitals. There were some few, but not many of the British wounded, taken prisoners, by no means so many as at Chippewa. Each side sustained fully its military renown, and each side claimed the victory. The regiments of the British line engaged in it have it still inscribed on their colors, according to the practice of their army on such occasions. By some it was considered as a drawn battle, and is still a subject of discussion in that respect in histories of that period. This battle was one of the most destructive field fights on record, for the numbers engaged.*

It crowded our hospitals so completely, that the attention of the surgeons was required unremittingly from early in the morning till night, besides the constant sick calls at night. At one time the author had the sole attendance and dressing of one hundred and seventy-three sick and wounded. My fingers became so sore from incessant dabbling in water and in pus, that I could seize nothing without pain, and was constantly liable to let articles fall, from the sudden twinges of agony in touching them.

The American army was so weakened by the battles of the 5th and of the 25th, that it retreated to Fort Erie, and there began to fortify itself. The British army, in the mean time, was strongly reinforced, and moving up began their operations against the position.

*Losses of opponents and their force on the field of battle, are, for the most part, conjectural, or founded upon reports through defective channels of information. Generally, losses on the hostile side, as well as amounts of force, are overrated on each side. The private official accounts rendered to the governments respectively, are the most accurate, because errors here might produce the most serious calamities in further operations.

Our pressure of hospital business continued till August the fourth, when an attack at Black Rock by a large force of the enemy, in view of capturing the munitions at Buffalo and the troops in the hospital, occasioned a sudden and general dispersion. Our requisitions for hospital rations for some days before this affair had been at eleven hundred, according to the steward's report.

The precise number of the wounded patients at this time, I have preserved no memorandum of, but it included a very large amount, as we had on hand those of both armies, somewhat intermixed; but the body of the British soldiers to themselves, in one division of the hospital. The wounded officers were generally quartered in the town, the soldiers being in tents.

The attack was made by twelve hundred men under Col. Tucker, and their operations were defeated by the gallantry of two companies of Riflemen under Major Morgan, who, having put up a breastwork of logs near the Conjocketa creek, a little below Black Rock, and removed the planks from the bridge crossing the creek, made his defence good at that point by cutting down the head of the British column as fast as it showed itself near the bridge. The fire was so destructive that the column would have been exterminated if it had persisted in its advance.

The danger of capture was so imminent, that while the action was in progress many of the patients of our hospital, who were capable of shifting for themselves, dispersed into the country, and others were removed to Williamsville, a small town of two or three hundred inhabitants, eleven miles in the interior.

At this place a general hospital was opened, and the writer placed in charge of the Buffalo one as a receiving hospital for the army now closely hemmed in at Fort Erie. The hospital at Williamsville had for its chief officers Drs. Ezek. Bull, W. Thomas, and T. Lovell. The latter was afterwards Surgeon General of the army, and had distinguished himself by his skill and zeal in the campaign of 1813, as well as in 1814, now going on. The hospital at Buffalo was directed to retain the most severe cases of wounds, and to send all others forward to the interior one, and to pursue this course for the remainder of the campaign. My care for the time was thus reduced to eighty or ninety, whose condition forbade removal. A physician in private practice in Buffalo, Dr. Coltrin, was allowed me as an assistant. He had been the partner in practice with Dr. Cyrenus Chapin, the oldest physician of the place. Dr. Chapin had been equally distinguished in his profession and by a military career. In the latter, he was Colonel of a regiment of volunteers, and had as such accomplished several remarkable feats of bravery, by his resistance to frontier attacks, by his hostile incursions into Canada, and by a singular re-capture of himself on Lake Ontario in an open boat, by rising, with some other American prisoners, upon his guard, and bringing boat and all into the American territory.

On the 15th of August, General Gaines being in command of the American forces, a general assault was made upon our works by the British General Drummond, the details of which are among the most stirring of the entire war. The British advancing in three columns before daybreak, two of these columns were repulsed with great slaughter. The third column, more successful, stormed the Fort, and while contending on a bastion for an hour for the final mastery, a magazine near it exploded and threw the column into the air ; this made the repulse decisive. Nine hundred and fifty men of the British, Mr. Ingersoll says, were killed, wounded, and made prisoners, about one-third of their entire force. The American loss was but eighty-four. It was one of the most brilliant events of the campaign, and gave imperishable renown to the General in command. The conflict lasted about three hours.

Those killed at once by the explosion, were estimated at from two to three hundred. On the 16th the survivors of the wounded, amounting to one hundred and forty-three, were brought from the Fort to our receiving hospital at Buffalo, and shortly afterwards ordered to the Williamsville hospital. They were truly most pitiable spectacles of the havoc of such a battle. Some blackened over the whole face with the explosion of the powder, and their heads swollen to two sizes ; some with eyes burned out ; in others, limbs mangled and perforated by musket balls, with their clothes torn from their scarified backs, &c., &c. There was scarcely a detail of wound which was not exemplified among them. This was the third great battle fought in fifty days from the opening of the campaign, and it alone was enough to show the horrors of war. It would be impossible to recall the precise impressions of that period, the military man sees in such events the steps of his glory, but the surgeon has only the impression of the woes of war ; of affrighted women and children leaving their homes precipitately to escape the bloody fury of an enraged enemy with his savage allies; he hears only the groans of the wounded, sees the horrid mutilation of their bodies, their want of comfortable accommodations and provisions, and the imperfect attendance from press of business. Since July the 2d, our hospital had been recruited by two pitched battles, one general assault upon our position at Fort Erie, by the defence of Black Rock under Major Morgan, and by a skirmishing, bombarding and cannonading, which had scarcely the interval of an hour from day to day.

These operations continued with but little interruption till Sept. 17th, when a sortie was made from Fort Erie. The besiegers were driven off from their works, and their cannon spiked or rendered useless, and three hundred and eighty prisoners were brought in. The American loss was five hundred and twenty-seven in killed, wounded and missing; the British loss six hundred and nine. Mr. Ingersoll says one thousand, nearly a fourth of their army. The sortie was a blow of consummate skill and daring, attended with the most heroic feats, in which General Peter B. Porter bore a conspicuous part. General Jessup considered it the most splendid achievement of the campaign.* This feat established the military fame of General Brown, who had resumed the command; and brought the campaign virtually to a close, as the subsequent part of it was comparatively uninteresting.

A final termination was put to the campaign on Nov. 5th, by the complete evacuation and blowing up of Fort Erie by the Americans. Offensive operations had now ceased to be thought of from the vast accession to the British forces ; and the attention of government was directed exclusively to the making our own territory secure. The British Commissioners at Ghent, at this period, had commenced with the prefatory and absolute demand,

*See for a detail of it, Ingersoll, loc. cit. p. 151.

that an immense belt of territory, coterminous with our northern frontier of lakes and rivers, must be given up, all Michigan, all Illinois, one third of Ohio, and the navigation to the ocean of the Mississippi. Other terms equally humiliating were mixed up with these. Washington had been captured, and everything indicated a recurrence of the scenes of the American Revolution. The government proposed a conscription of one hundred thousand men to begin with for the next campaign. Happily more moderate views finally dictated the terms of peace, and the victory of New Orleans under General Jackson ratified them in the judgment of both parties.

After the blowing up of Fort Erie, by the American garrison consisting of Col. Hindman's command of Artillerists, the author was placed in charge of them, with great pleasure to himself, owing to the high renown they had acquired. This battalion had so distinguished itself during the whole of the campaign, as to attract even the commendation of the British officers, who had got the impression that it was under the direction of the most experienced French artillerists. They were quite surprised on learning that the officers were all Americans, and none of them beyond thirty years of age.

On the 23d of December the General Hospital was closed at Buffalo, and the patients sent to Williamsville. Being thus freed, my service for that campaign closed, and I left the station for Washington on the 24th, having in hopes promotion for the next campaign, as we then knew nothing of the pacific tone which the negotiations at Ghent had assumed.

From the preceding narrative, it will be inferred that every description of wound was to be met with, as from the musket ball, from the grape shot, cannon ball, fragments of shell—in fact all the missiles used in warfare. There were but few instances on either side of the bayonet wounds, as troops seldom close so much as to inflict them.

Men who have witnessed the conduct of others during protracted scenes of danger, are struck with the great disregard of life which seems to infuse itself into their habits. Man is the only animal who, from being naturally cowardly and afraid of pain and danger, gets so as to disregard them both. Dr. Mann (Medical Sketches, p. 175,) has remarked justly, that " long abstinence, watchings and unremitted hardships, soon break down not only the spirits but strength of an army. But when well fed, the men cheerfully endure fatigue and cold, and expose themselves to the most threatening dangers, regardless of consequences. Familiar with death, the soldier soon forgets that the feeling of horror was once attached to its name. The love of country, honor, the pride of conquest, incite him to acts of heroism. When duty calls to confront the enemy, he obeys the summons with the same alacrity as when invited by the alluring voice of pleasure to his amusements." Even female followers of an army fall into this mood. Among us was one from Kentucky, remarkable for her height, muscular figure, for the loss of one eye, and for her volubility in oaths and queer modes of execration when jeered at or incensed. In the preceding year, her father and brother had been among the victims of a celebrated but execrable massacre at the River Raisin, of which she was witness. Storming with feelings of revenge, at the opening of this campaign, Betsey, dressed as a soldier, entered the ranks, and at the battle of Chippewa executed her firing with the precision of one of the line. Her company was much exposed, and her immediate comrades shot down; she nevertheless continued in line, until the Captain told her it was time to leave, that the wounded men required water and attentions, and that she had better serve them. She obeyed, came into Hospital with them, and was one of the most faithful and kind of nurses, notwithstanding her recklessness of conduct in other respects.

I remember, one day, in making my hospital rounds, a patient just arrived presented an amputated forearm, and in doing so could scarcely restrain a broad laugh; the titter was constantly on his face. "What's the matter? this does not strike me as a subject of laughter." "It is not, Doctor, but excuse me, I lost my arm in so funny a way, that I still laugh, whenever I look at it." "What way." "Our first Sergeant wanted shaving, and got me to attend to it, as I am a Corporal. We went out together in front of his tent, I had lathered him, took him by the nose, and was just about applying the razor, when a cannon ball came, and that was the last I saw of his head and of my hand. Excuse me, doctor, for laughing so; I never saw such a thing before." This occurred during the siege of Fort Erie.

Out of barracks it is common for messes of soldiers to cook at fire places made of two banks of turf, crossing at right angles like the ridges of the occipital bone. When not on parade, these places are the resort of groups of soldiers. On an occasion of the kind, one of the soldiers, standing on one foot, a cannon ball struck him on the head, and in doing so gave a whirl to the whole body upon the leg as he stood; the other leg flew out, as the headless trunk turned, and it upset a camp kettle of soup in the process of cooking. The soldier to whom it belonged was quite indignant at the loss, (provisions were then very scarce at the Fort,) and in his wrath he ejaculated, "could you not have lost your head without kicking over my soup?"

A patient who had lost his entire scalp to a level with the top of his ears, was the subject of unmitigated jeers by his comrades. Owing to the profusion of the suppuration, he was brought out daily upon the parade in front of the hospital tent, to have his wound washed and dressed. As there was some degree of regularity in the hour, it constantly attracted a circle of the more lightly wounded. It appeared that he had met with his accident from dropping behind to pick his flint, as he said, but it occurred as his regiment was moving forward into action, and the belief was that his motive was to save himself, instead of improving his flint. A party of hostile Indians on scout in the rear of the regiment, rushed upon him, he was felled with the butt of a tomahawk, and his scalp immediately stripped off in a large circular cut. He would after this have got the *coup de grace* from the edge of the tomahawk, but he feigned death; at this critical moment a scouting party of Americans fired upon the band of Indians, and they scampered away.

The battle being over, the wounded of the regiment were surprised to find their comrade alive, as he was considered a victim to the Indians. But the whole affair being now understood, it was a constant subject of merriment as his denuded cranium was exposed for the surgical dressing. One would ask him who cut his hair; another, how long it took; another declared that if the Indians were such close cutters, they should never touch his hair. And so it went on from mouth to mouth, each soldier trying his ingenuity at a question, as to what the wounded man had said on the occasion, how he managed it, and what the Indians said.

The crowd of wounded brought in by the field of Bridgewater reduced unavoidably attentions to individuals. The most possible was done, but it did not come up to the point. Under these circumstances many soldiers were treated in the outskirts of the hospital, by their wives or females having an attachment to them. With such assiduities, recoveries took place which would scarcely have followed in the ordinary hospital practice. A boy shot in the forehead, with the ball penetrating to the back of the neck along the base of the face, whom I had seen on his first arrival and dressed, and given up for a fatal case, I was pleasantly surprised some weeks after in finding in a state of convalescence. A soldier, shot through the lungs badly, was saved in the same way. So much for assiduous nursing, congenial food, cleanliness and good poultices.

(To be continued.)

